# Parental anxiety and form of parenting during the COVID-19 pandemic

**DOI:** 10.1186/s40723-022-00103-2

**Published:** 2022-10-11

**Authors:** Elfan Fanhas Fatwa Khomaeny, Erika Setyanti Kusumaputeri

**Affiliations:** 1 Faculty of Teacher Training and Education, Muhammadiyah University of Tasikmalaya, Tamansari street km. 2,5, Tasikmalaya, West Java Indonesia; 2 Sunan Kalijaga State Islamic University, Yogyakarta, Indonesia

**Keywords:** Anxiety, Parents, Forms of parenting, COVID-19 pandemic

## Abstract

The massive development of information technology based on big data, internet, and artificial intelligence has brought fundamental changes to human patterns and lifestyles, especially after the COVID-19 pandemic that hit globally, has added to a large and complex problems in parenting, as well as demanding people to take care of their children. Parents must be able to adapt and reposition themselves with new and effective forms of parenting, this can increase parental anxiety. To determine the level of parental anxiety, this research was conducted using a quantitative descriptive method through the distribution of questionnaires based on the GAD-7 instrument. This study focuses on efforts to capture the level of parental anxiety and the need for a new form of parenting. The results can be the basis for further research to find and develop new forms of parenting. The results of research on 669 parents living in West Java, Indonesia, showed that the level of parental anxiety was 63.08% at the level of moderate and severe anxiety. The level of parental satisfaction regarding the form of parenting used is at a low level of 67.12%, while the level of parental interest in the new form of parenting is at a very high level of 98.51%. The need for the latest form of parenting that can respond to the challenges and demands of the times is very necessary to minimize parental anxiety.

## Introduction

The massive development of information technology based on big data, internet, and artificial intelligence has brought revolutionary changes to human patterns and lifestyles. Today's life is characterized by very fast changes, sudden changes, many surprises, and unfriendly to slow ones, so this era is called the era of the Industrial Revolution 4.0 (IR-4.0).

The era of IR-4.0 which is marked by advances in information and communication technology is basically an achievement of the progress of modern human science as an answer to the problem of reality. Science and technology were created by humans to provide the best solutions to many problems faced by humans, which from time to time are always changing. According to Thomas S. Kuhn, author of The Structure of Scientific Revolution, the advancement of ICT in the IR-4.0 era is a form of revolutionary science (Nur Kholik et al., 2020).

In addition, the era of IR-4.0 not only brings up many advantages and positive things for human life, but also has led to many losses and negative impacts on human life, including: the sophistication and practicality of human life will encourage humans to be selfish and individualistic; the law of the jungle will become the prevailing law, where the strong are the ones who rule, and the weak are controlled and oppressed or more precisely, the modern version of slavery will return; Human dependence on the internet and its supporting tools is very high, when there are disturbances or obstacles to it, humans cannot do anything; levels of stress and technology addiction increased dramatically; the norms and values ​​of religion or morality in human life are weakened; the ability to socialize directly becomes low and rare; the existence of human physical vulnerability; low social emotional and prone to conflict and confrontation; there will be irregularities in human life and everything becomes difficult to predict, because everything is fast-paced and can change suddenly; the loss of linguistic and cultural diversity, because what exists is international language and culture, while local culture will be eroded and degraded slowly, either because of cultural assimilation or because the language and culture is abandoned by humans themselves (Chandrawaty & Khomaeny, 2019).

The era of IR-4.0 had a tremendous impact on fundamental changes to current patterns and human lifestyles, both individually and socially. Changes in human patterns and lifestyles have automatically changed the challenges that must be faced by humans, which require humans to make major changes in response to any changes that occur, humans can still adapt, reposition themselves, and maintain their existence.

The changes mentioned above also have a big impact on the parenting and educational process for children. Parents must try to find the right and correct parenting pattern for the millennial generation or the digital native generation. On the other hand, parents as a digital immigrant generation experience many obstacles, because there is a large gap in the use and mastery of the digital, especially their parents' past life experiences that have influenced and shaped their mindset and behavior.

Developments and changing times have demanded parents to adapt and reposition themselves in the use form of parenting for their children, which is increasingly difficult to predict and changes very quickly, especially with the outbreak of the COVID-19 pandemic that has hit the global community, added the problem become large and complex for parenting, and caused the level of parental anxiety increase. On the other hand, the development and discovery of effective forms of parenting that can be used by parents in caring for their children does not go fast, even shocks the community with the events they face, and parenting becomes unclear because of the additional new burden for parents who have to educate their children at home, because almost all schools in the world are closed and it greatly impacts nearly 1.6 billion children in 190 countries (De Giusti, 2020) in all aspects of children's lives (Barnard, 2020; Di Pietro et al., 2020).

The COVID-19 pandemic does not only have a direct impact on children as objects of parenting, but also on parents who are the subject of parenting itself. The increasing new burden for parents who have to care for their children at home has increased pressure on parents, it has an impact on stress and increases parental anxiety (Cluver et al., 2020; Wu & Xu, 2020).

Several research results on parental anxiety during the COVID-19 pandemic showed that almost one of three parents or 35.7% of respondents studied had a severe level of anxiety (McCormack et al., 2020). In the United Arab Emirates, almost 71% of population reported having anxiety, which 38% reported being at moderate and severe levels of anxiety (Saddik et.al., 2020). Even parents who know that their acquaintances have been exposed to COVID-19, they have a high level of anxiety, based on research being diagnosed as having a high anxiety level reaching 57.61% (Akgul et.al, 2021). The high level of parental anxiety also affects the decision to get health service assistance. 65.0% of parents with moderate and severe levels of anxiety were influenced in deciding to get health service assistance, but it was not correlated with vacation planning decisions (Kwiatkowska, et.al, 2021). The level of parental anxiety during the COVID-19 pandemic is very influential on parental attitudes associated with potential children abuse, where great support and ability to control the perception of the COVID-19 pandemic is closely related to low levels of stress and potential children abuse (Brown, 2020).

The research above focuses on the impact of the COVID-19 pandemic on parents and children, but there is no visible effort by researchers to provide a discourse on the need for new breakthroughs related to the latest forms of parenting that can adapt to the changes and demands of today's era. This study was conducted to find out how the level of parental anxiety about the future of their children during the COVID-19 pandemic, and the level of parents’ interest in a new form of parenting that can overcome their anxiety.

The form of parenting cannot be separated from the past experiences of parents and the fear that arises due to the facts and reality experienced or witnessed encourages parents to make assumptions, predictions and projections for the future. This gives the parents a sense of concern on the future of their children. Parenting style will greatly affect the optimization of children's growth and development achievements, including: academic ability (Dehyadegary et al, 2012; Rivers, 2008; Sadiq, 2018; Theresya et al., 2018; Yang & Zhao, 2020; Zahedani et al, 2016), social emotional skills (Berg, 2011; Morris et al., 2017; Nezhad et.al, 2015), attitude/behavior and moral (Charalampous et al., 2018; Hawkins, 2005; Lee et al., 2018; Loudová & Lašek, 2015; Rosli, 2014), confidence (Fuentes et al, 2019; Lynn & Ting, 2019; Martinez et al., 2020), creativity (Mehrinejad et al., 2015), and children welfare (Bahrami, 2017).

The form of parenting are fundamentally based on two approaches, namely, warmth and supervision by parents for their children, this can be broken down into 6 dimensions in determining The form of parenting, namely: warmth, rejection, structure, chaos, autonomous support, and coercion. (Skinner et.al, 2005). The forms of parenting include: *Permissive/Indulgent parenting*, *Authoritative parenting*, *Authoritarian parenting*, and *Uninvolved parenting* (Baumrind, 1967; Darling, 1999; Kuppens & Ceulemans, 2019; Richfield, 2017; Robinson et al., 1995).

Along with the changes and developments of the times, the forms of parenting are built on the basis of fear and anxiety of parents about the future of their children, and those have given rise to various new forms of parenting, namely: Neglectful parenting (Gaudin, 1995); Positive parenting (Neighbourhoods, 2020); Narcissistic parenting (Cohen, 1998; Dentale et al, 2015; Evans, 2018; Kenyon, 2020; Watson et al, 1992); Overparenting or Helicopter parenting (Earle & LaBrie, 2016; Hesse et al, 2018; Lemoyne & Buchanan, 2011; Odenweller et al, 2014; Winner, 2019); Slow and steady parenting (Sanderson, 2007); Toxic parenting (Dunham et al, 2011; Forward & Buck, 1990); Dolphin parenting (Kang, 2015); Hypnoparenting (Firdaningrum et.al, 2019; Wasmin et al, 2019); Hyperparenting (Jansen, 2015; Venkatesan, 2019) Tiger parenting (Chua, 2011; Fauziyah, 2020; Fu & Markus, 2014; Kim et al, 2013); Elephant parenting (Kroll, 2004; Musman, 2020); Lighthouse parenting (Byrne et.al, 2019); Spiritual parenting (Anthony, 2010); Unconditional Parenting or Conscious Parenting (Cousens & Lynn, 2015; Plugarasu, 2020; Rahmqvist et al, 2014); Jellyfish parenting, Brickwall parenting, Backbone parenting (Coloroso, 2010); Free range parenting (CBC Pimentel, 2016; Radio, 2013); Punitive parenting (Zubizarreta et al, 2019); Islamic Parenting (Akin, 2012; Rahmawati, 2016; Ubaidillah, 2019; Yani, 2017); Prophetic Parenting (Hairina, 2016; Suwayd, 2010); Kingdom parenting (Munroe & Barrows, 2011); Christian Parenting (Sinclair, 1992); Jewish Spiritual parenting (Kipnes & November, 2015); Intuitive parenting (Goode & Paterson, 2009; Snyder, 2010); Sacred Parenting (Glickman, 2009; Thomas, 2017, 2018); Mindful parenting (Race, 2014; Rogers, 2005; Bögels & Restifo, 2013); Digital parenting (Maisari & Purnama, 2019; Ulfah, 2020; Wong et al, 2020); Screen Smart parenting (Gold, 2014); Cyber ​​Smart parenting (Primary, 2012); Indonesian Parenting (Khomeny et al., 2020); Parenting with Heart (James & Dodd, 2018; Phelan & Webb, 2018); Parenting with Love (Bienenfeld, 2014; Anshor & Ghalib, 2010); Adaptive parenting (Claudio, 2016; Osofsky & Thompson, 2000; Prakoso, 2018); Enlightening parenting (Fitriani, 2017); Screaming Free Parenting (Perdana, 2011; Runkel, 2008); The Danish waf of parenting (Alexander & Sandahl, 2018); Islamic Hypnoparenting (El Shakir, 2014), and there are many forms of parenting that exist, including the latest parenting pattern associated with technological advances as revealed by Sun Sun Lim, namely, Transcendent Parenting (Lim, 2019; Livingstone & Ross, 2020).

The form of parenting will continue to develop and create new forms of parenting, which are adapted to the challenges and changing times and referring to the main parenting functions, namely: efforts to control children behavior, children psychology, building emotional warmth, media for transformation values, beliefs and culture, the process of stimulating and educating for their children (Cerezo et al, 2018; Kilonzo, 2017; Power, 2013; Skinner et al, 2005; Smetana, 2017).

New forms of parenting will continue to emerge along with the level of parental anxiety, because parents always want the best for their children, and most parents are willing to sacrifice their fun and are willing to spend all their power and effort, both material and immaterial for the success and happiness of their children. Research on parental anxiety and forms of parenting during COVID-19 pandemic has been widely carried out, predominantly related to the impact and the efforts can be made to overcome these impacts as focusing on the impact on children and family life in general.

This article more specifically describes the level of parental anxiety. The brief description of the satisfaction and importance of a new form of parenting in accordance with the challenges and demands of the times, aims to strengthen the results of research related to anxiety, it can describe the anxiety of parents towards their children comprehensively, and can be the basis for further research related to new forms of effective parenting and can minimize parental anxiety.

This research has significance as a basis for researchers and educators to develop and find new forms of effective and contemporary parenting, to minimize parental anxiety about the future of their children. The facts, the times are changing so fast, but the development of research on parenting is very slow.

The problem that will be studied is how the level of parental anxiety during the COVID-19 pandemic, to strengthen the results of the study, it is also necessary to examine how the level of satisfaction of parents with the form of parenting they have used, and the level interest of parents with the new form of parenting.

The COVID-19 pandemic can increase the level of parental anxiety about the future of their children, so that it will also have an impact on the level of interest of parents in new forms of parenting that can overcome their anxiety. Therefore, what is the level of anxiety and the level of interest of parents in caring for children during the COVID-19 pandemic?

## Research methods

### Participants

Participants in this study amounted to 669 parents living in all districts in West Java, with details of 551 female participants and 118 male participants. Using snowball sampling, the data was initially collected from respondents living in districts in East Priangan, then increased to the level of the province of West Java.

### Instrument

The instrument in the questionnaire consists of 15 questions that must be answered by the respondent, consisting of: 6 questions related to the identity of the respondent, 2 questions related to the level of satisfaction with the form of parenting they have used and the level of interest in the latest parenting patterns that are in line with the challenges of the times. To determine the level of parental anxiety associated with parenting patterns, using an instrument that refers to Robert L. Spitzer's theory, namely: Generalized Anxiety Disorder (The GAD-7), which consists of 7 instruments, namely: Feeling nervous, anxious or on edge; Not being able to stop or control worrying; Worrying too much about different things; Trouble relaxing; Being so restless that it is hard to sit still; Becoming easily annoyed or irritable; and Feeling afraid as if something awful might happen (Spitzer et al, 2006).

Based on the foregoing, the question instruments included in the questionnaire are as follows:

1. I feel anxious when imagining the future of my children, when associated with changes and developments of the times.
2. I can't control my feelings of worry about my children future.
3. I'm worried about the many differences due to the changes and development of the times.
4. I can't rest in peace when imagining my children future.
5. I'm nervous when I imagine the future of my children.
6. I'm easily offended when someone underestimates the future of my children.
7. I'm afraid that something bad will happen to my children in the future.

The research instrument used in the study has been examined and obtained ethical approval from the Ethics Committee of the University of Muhammadiyah Tasikmalaya. Before the respondents filled out the questionnaire, they received an explanation and were asked for their consent.

### Procedure

This study used a questionnaire through the google form application, which was distributed randomly to respondents who have become parents and live in districts in the province of West Java, Indonesia. Questionnaires were distributed through the WhatsApp group network, each respondent was only given the opportunity to fill out the questionnaire once, and was not given access to refill the questionnaire. The data collection of the results of the questionnaire was carried out on January 3–7, 2021.

### Data analysis

all data from the Respondents answer was verified, then coded before data analysis was carried out. Each answer on the questionnaire instrument was assessed and converted using a Likert scale, as follows: 0 for the answer never; number 1 for the answer sometimes; number 2 for frequent answers; and number 3 for the answer always. The total score of each respondent is categorized according to the following scoring criteria (Table 1).

**Table 1:** Parental anxiety level assessment criteria

No.	Score	Anxiety level
1	0–4	Minimum Anxiety
2	5–9	Mild Anxiety
3	10–14	Moderate Anxiety
4	15–21	Severe Anxiety

## Results and discussion

### Result

#### Validity test and reliability test

The research instrument has been tested for the level of validity. The results of the Pearson Product Moment validity test, all instruments have an r-count above the r-table both r-table with a significance of 5% and 1%, so that the instrument can be categorized as a valid instrument, as shown in Table 2.

**Table 2:** Validity test results

No.	Instrument	r-Count	r Table	Conclusion
1	X_1_	0,719	0,105	Valid
2	X_2_	0,783	0,105	Valid
3	X_3_	0,770	0,105	Valid
4	X_4_	0,793	0,105	Valid
5	X_5_	0,832	0,105	Valid
6	X_6_	0,727	0,105	Valid
7	X_7_	0,799	0,105	Valid

The instrument was also tested for the level of reliability. The results of the reliability test of Cronbach's alpha on the instrument is 0.89, because it is at a value of 0.9 > α ≥ 0.8, then the internal consistency of the instrument can be categorized as a good instrument.

#### Parental satisfaction and interest level

**Fig. 1:**
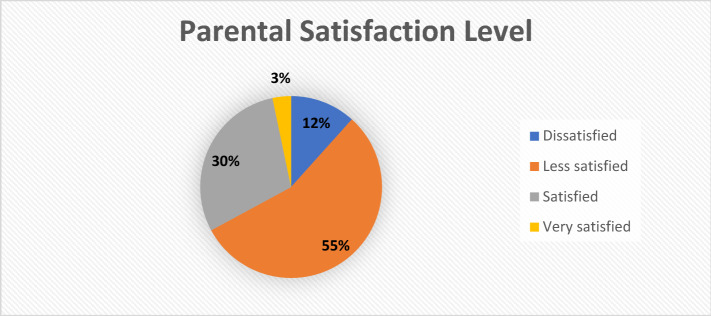
Parental satisfaction level chart

A measurement of the satisfaction and interest of parents in the new form of parenting is carried out to strengthen the results of research related to parental anxiety. Photographing the level of satisfaction of parents regarding the form of parenting that has been used to care for and educate their children, is not carried out in detail but only in general terms. From 669 respondents, who stated that they dissatisfied were 78 parents or 11.66%, less dissatisfied were 371 parents or 55.46%, satisfied were 198 parents or 29.60%, and very satisfied were 22 parents or 3, 29%, as illustrated in Fig. 1.

**Fig. 2:**
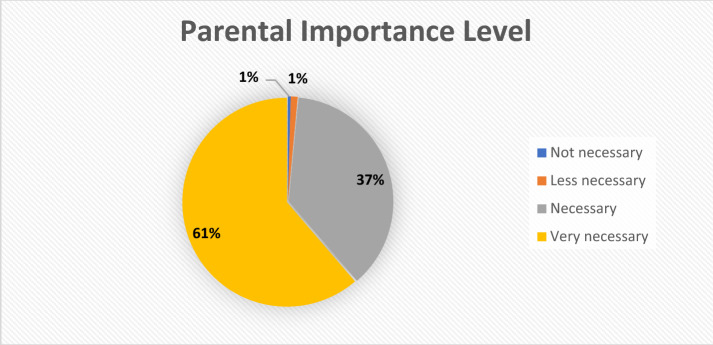
Parental importance level chart

Based on this research, 67% of parents are not satisfied with the form of parenting they use. These are because the challenges and demands are very dynamic and directly proportional to the changes in behavior and psychology, and the approach used by parents must also be updated. This also has opened up a wide space to find and offer new forms of parenting are able to answer the challenges and demands of the times. The interests of parents in a new form of parenting can respond to the challenges and demands of the times can be described in Fig. 2.

There were 3 parents or 0.45% of parents who stated it was not necessary, 7 parents or 1.05% of parents who stated it was less necessary, 249 parents or 37.22% of parents who stated it was necessary, and 410 parents or 61.29% of parents who stated it was necessary. Based on the research data, 98.51% of parents really need the latest form of parenting that can answer the challenges and demands of the times, this also reflects that parents' anxiety is still very high.

#### Parental anxiety level

Excavation of information about parental anxiety level in parenting was conducted to describe the level of satisfaction of parents about the form of parenting they use and the parental interest level about the new form of parenting. The dynamic developments and changes of the times will greatly affect the behavior and psychology of children. Thus, demanding a change and updating the form of parenting does not only focus on how to answer the problems that exist today, but also can prepare children to be able to exist and have the ability to answer future problems.

Projections or predictions of future challenges and demands will be very much different from today's, we can see this one of them with the parameters of today's information technology developments. Today's information technology is based on big data, internet, and artificial intelligence, which will massively provide fundamental changes to human patterns and lifestyles.

**Fig. 3:**
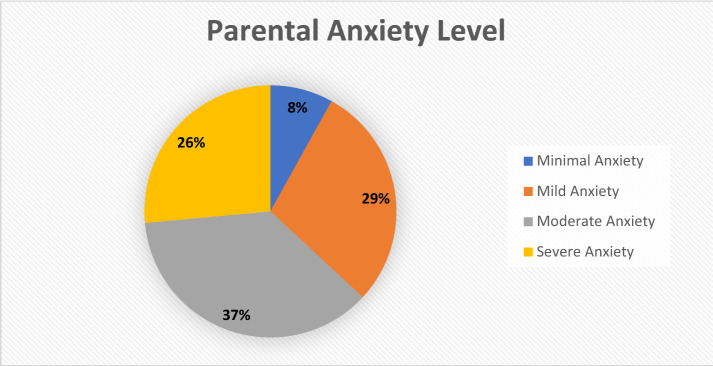
Parental anxiety level chart

The form of parenting cannot be separated from the past experiences of parents, and it arises because of fear which comes based on facts and realities experienced or witnessed by the parents that encourage them to make assumptions, predictions and projections of the future. This gives parents a sense of concern on about the future of their children. Based on research on parental anxiety using the instrument from Robert L. Spitzer, namely: the Generalized Anxiety Disorder (The GAD-7) instrument is described in Fig. 3.

After analyzing the data, from 669 respondents who filled out the questionnaire, it was found that 54 people (8.07%) were categorized with minimal anxiety or had no anxiety, 193 people (28.85%) were categorized with mild anxiety or had low anxiety, 245 people (36.62%) were categorized with moderate anxiety or had moderate anxiety, and 177 people (26.46%) were categorized with severe anxiety or had high anxiety. Based on these data, 63.08% of parents have anxiety above normal, if it cannot be managed and anticipated properly, it will have the potential for anxiety disorders. The following are the results of a detailed study of parental anxiety based on the classification of Gender, Educational Background, Age, Income, Occupation, and City/Regency of the respondent's residence (Table 3).

**Table 3:** Parental anxiety levels based on gender, educational background, income, occupation, and the district, where the respondent lives

No.	Respondent identity	Anxiety level			
		Minimum anxiety	Mild anxiety	Moderate anxiety	Severe anxiety
A	Gender				
1	Female	6.90	27.04	37.21	28.86
2	Male	13.56	37.29	33.90	15.25
B	Educational background				
1	Elementary School	6.25	12.5	25.00	56.25
2	Junior high school	2.17	30.43	26.09	41.30
3	High school	4.32	17.28	44.85	33.55
4	College	12.75	40.85	30.72	15.69
C	Age				
1	20–30 Years	4.76	19.05	42.86	33.33
2	31–40 Years	10.49	33.57	32.17	23.78
3	41–50 Years	4.17	26.79	41.07	27.98
4	> 50 Years	14.71	35.29	30.88	19.12
D	Income				
1	< 2,000,000	4.93	22.66	40.39	32.02
2	2,000,000–3,999,000	14.75	32.79	33.61	18.85
3	4,000,000–5,999,000	10.45	53.73	22.39	13.43
4	> 6,000,000	12.28	38,60	31.58	17.54
E	Profession				
1	Housewife	5.84	20.95	39.26	33.95
2	Government employees	17.39	41.30	26.09	15.22
3	Private employees	10.92	42.53	29.89	16.67
4	entrepreneur	4.76	31.75	47.62	15.87
5	Others	22.22	11.11	33.33	33.33
F	City/District				
1	Tasikmalaya City	8.30	33.19	34.93	23.58
2	Tasikmalaya Regency	4.15	21.45	43.60	30,80
3	Other Cities/Districts	15.23	36.42	25.83	22.52

Based on the data above, when using a measure of the level of anxiety at a severe anxiety level, then female have a higher level of anxiety than male. The level of parental education also affects the level of parental anxiety. The low level of parental education has an impact on the high anxiety of parents. Conversely, the high level of parental education has an impact on the lower parental anxiety.

The level of parental anxiety is influenced by the age of the parents. parents with old age, have lower anxiety levels, although based on this study, in parents with an age range of 41–50 years, there is an increase in parental anxiety levels, although the increase is only about 3% but it is still very low compared to parents' anxiety levels with an age range of 20–30 years. Parents' anxiety levels are also influenced by parental income, parents with high incomes have lower anxiety levels.

The level of anxiety of parents based on their profession is described as follows: parents who work as housewives have a higher level of anxiety than working mothers (employees and entrepreneurs). The anxiety of housewives is at the level of 33.95%, while the anxiety of government employees, private employees and entrepreneurs is at the level of 15–17%. The level of parental anxiety based on the district, where the parents live, based on the results of a questionnaire from parents in 24 District in West Java, Indonesia, shows that the average level of parental anxiety is at level 22–30%.

The results of the parental anxiety study above, strengthen the results of research on the level of parental satisfaction with the form of parenting used and the level of parental interest in the latest and latest forms of parenting, which are able to answer the challenges and demands of the times, and are oriented towards preparing children to future.

## Discussion

The direct impact of the COVID-19 pandemic will decrease and disappear in line with the disappearance of the outbreak, but the indirect impact will still affect family life, including the impact of using digital media in various family activities, especially in early childhood. The development and massive use of the internet, big data and artificial intelligence in the family, has offered information and communication services that are always on and available 24 h a day. these services shape family communication practices and media consumption habits, influence how parent guide their children's media use, and how parents and children can connect with one another (Lim, 2016).

Always-on parenting, where the time for communication becomes round the clock via technology-mediated connectivity. Parents must be prepared to face endless digital connectivity and the task of parenting no longer exists only when parents and children are together (Lim, 2018). Changes in patterns and relationships between family members facilitated by digital media have become a new challenge and demand for parents in the parenting process.

The use of digital media in parenting cannot be separated from parental anxiety about negative impacts and risks for children such as the risk of being exposed to violent media content, sexuality and pornography, drug abuse, biased beauty standards, the influence of political extremism (Genner & Suss, 2017). the potential of media that can damage moral values ​​and the ability of the media to trigger fear of moral threats (moral panic) in children's social environment (Tamborini et.al., 2017). The term moral panic is used to describe a broad spectrum of social phenomena that culminates in social policy and reactions in many areas, such as: global warming, immigration, asylum seeking, hunting for political criminals, drug abuse, rampant crime, juvenile delinquency, child sexual abuse., trafficking in women, football hooliganism, mugging, sex, acts of terror, drug use in sports, satanism, and many more (Yehuda, 2017).

The COVID-19 pandemic encourages parents to tend to experience anxiety about the excessive use of media. Parents anxiety as a reaction to social, economic, and technological changes have encouraged parents to devote all their intellectual and emotional energy, to find an appropriate parenting concept or educational institution that can provide the best care and education for their children (Cucchiara, 2013).

When parents are faced with the choice to embrace, balance or resisting digital media in their family life (Livingstone & Ross, 2020), making a choice is not an easy that can be decided based on considerations of motivational aspects only, but includes various aspects, such as: the parents readiness to know and be skilled in using digital media, financial readiness to purchase appropriate and safe digital media for children, parents and children readiness to be bound by a regulation or mutual agreement regarding the use of digital media, because the digital world has become a field, where families, especially parents, negotiate identities, relationships, values, and life opportunities for their children.

This study seeks to motivate and encourage academics and early childhood education practitioners to develop and find new forms of parenting that are adaptive, dynamic and transcendent as a solution to overcome parental anxiety and become a reference for parents in making decisions in responding to the use of digital media in their families, parents can carry out their main function as parents, namely, guiding and supervising the use of digital media by children, to ensuring the use of digital media is safe and has a positive impact on family life and relationships. This study has limitations, because the research sample only covers a few cities/districts in West Java, Indonesia.

## Conclusions

The level of parental anxiety will affect the level of satisfaction and parent interest about the form of parenting that is being or will be used to care and educate children. If the level of parental anxiety is high, then the level of parental satisfaction about parenting is low, and the level of parental interest in the latest form of parenting is high.

Based on a literature study on the types and forms of parenting, researchers found more than 40 forms and names of parenting, and it is predicted that new forms of parenting will continue to emerge that are adapted to the challenges and demands of the times. Moreover, based on the results of the research above, that the level of parental anxiety in West Java is at the level of 63.08%, referring to the criteria for moderate anxiety and severe anxiety levels leads to abnormalities. The level of parental satisfaction with the form of parenting they use is at a low level of 67.12%, referring to the criteria for being dissatisfied and less satisfied, while the level of parental interest in the new form of parenting is at a very high level of 98%.

This research can be used as a basis for other researchers to develop and find forms of parenting that are effective, contemporary, and responsive to developments and changing times, they can provide alternative forms of parenting. Research that reveals the impact of the COVID-19 pandemic is not enough, but must arrive at research on solutions that parents can use in dealing with the impact of the COVID-19 pandemic on parenting.

This research can be used as a warning for parents to adapt to the changes and demands of the times, by increasing knowledge and life skills in various situations, even in difficult conditions such as the COVID-19 pandemic which has an impact on the level of parental anxiety due to unpreparedness and incompetence. Academics and practitioners of child education must also continue to seek new forms of parenting that are adaptive, dynamic and transcendent.

## Data Availability

The data sets generated or analyzed during the current study are not publicly available due to maintaining respondent privacy but are available from the corresponding author on reasonable request.

## Abbreviations

IR-4.0: The Industrial Revolution 4.0
The GAD-7: Generalized Anxiety Disorder which consists of 7 instruments
ICT: Information and Communication Technology
